# FROM THE PERSPECTIVES OF PROVIDERS TO OLDER ADULTS: CONVERSATIONS ABOUT BEHAVIORAL HEALTH SERVICES IN OREGON

**DOI:** 10.1093/geroni/igae098.4196

**Published:** 2024-12-31

**Authors:** Ellis Hews, Dani Himes, Allyson Stodola, Sarah Dys, Walter Dawson

**Affiliations:** Portland State University, Portland, Oregon, United States; Portland State University, Portland, Oregon, United States; Portland State University, Portland, Oregon, United States; Portland State University, Portland, Oregon, United States; Oregon Health & Science University, Portland, Oregon, United States

## Abstract

The Older Adult Behavioral Health Initiative (OABHI) is an innovative program funded by the Oregon Health Authority to address gaps in services for older adults and people with physical disabilities with behavioral health (BH) issues. The Initiative consists of 24 Behavioral Health Specialists who work with community partners across Oregon to improve BH services through workforce development, health and wellness promotion, complex care consultations, and promoting coordination and collaboration between aging and BH agencies. The Institute on Aging (IOA) at Portland State University (PSU) has evaluated the effectiveness of the Initiative since its establishment in 2015. Prior evaluation efforts included interviews and surveys from key community partners, staff from Area Agencies on Aging (AAA), Aging and Disability Resource Centers (ADRC), Aging and People with Disabilities (APD), and Community Mental Health Programs (CMHPs). As the Initiative comes upon nearly ten years of existence, the evaluation team conducted a new evaluation approach by facilitating focus groups with these key community partners in addition to primary care providers, older adults (65+) with lived experience, and senior centers representing 25 rural and urban Oregon counties (n=54). Participants were asked about the top challenges in their communities and how the Initiative can better address their biggest needs when supporting older adults and people with physical disabilities living with behavioral health issues. Findings from these data identify recommendations to address systems-level barriers and opportunities to promote intra-agency collaboration and outreach to community partners and consumers.

